# Highly Efficient Photocatalyst Fabricated from the Chemical Recycling of Iron Waste and Natural Zeolite for Super Dye Degradation

**DOI:** 10.3390/nano12020235

**Published:** 2022-01-12

**Authors:** Fatma Mohamed, Safwat Hassaballa, Mohamed Shaban, Ashour M. Ahmed

**Affiliations:** 1 Nanophotonics and Applications (NPA) Lab, Physics Department, Faculty of Science, Beni-Suef University, Beni-Suef 62514, Egypt; f_chem2010@yahoo.com (F.M.); ashour.elshemey@gmail.com (A.M.A.); 2 Polymer Research Laboratory, Chemistry Department, Faculty of Science, Beni-Suef University, Beni-Suef 62514, Egypt; 3 Department of Physics, Faculty of Science, Islamic University in Madinah, Al Madinah Al Munawwarah 42351, Saudi Arabia; safwat.hassaballa@iu.edu.sa

**Keywords:** rusted iron, Fe_2_O_3_-zeolite photocatalyst, methylene blue, photodegradation

## Abstract

In this paper, Fe_2_O_3_ and Fe_2_O_3_-zeolite nanopowders are prepared by chemical precipitation utilizing the rusted iron waste and natural zeolite. In addition to the nanomorphologies; the chemical composition, structural parameters, and optical properties are examined using many techniques. The Fe_2_O_3_-zeolite photocatalyst showed smaller sizes and higher light absorption in visible light than Fe_2_O_3_. Both Fe_2_O_3_ and Fe_2_O_3_-zeolite are used as photocatalysts for methylene blue (MB) photodegradation under solar light. The effects of the contact time, starting MB concentration, Fe_2_O_3_-zeolite dose, and pH value on photocatalytic performance are investigated. The full photocatalytic degradation of MB dye (10 mg/L) is achieved using 75 mg of Fe_2_O_3_-zeolite under visible light after 30 s, which, to the best of our knowledge, is the highest performance yet for Fe_2_O_3_-based photocatalysts. This photocatalyst has also shown remarkable stability and recyclability. The kinetics and mechanisms of the photocatalytic process are studied. Therefore, the current work can be applied industrially as a cost-effective method for eliminating the harmful MB dye from wastewater and recycling the rusted iron wires.

## 1. Introduction

Iron has long been considered a vital industrial material due to its vast variety of applications. Millions of tons of rusty iron waste every year are produced as a result of the corrosion in the air, resulting in environmental pollution and economic losses. Therefore, the reuse/recycling of rusty iron wire is a good method to decrease the wastes amounts. The corrosion of iron wires in the atmosphere produces a large amount of rust iron every year. This leads to the formation of millions of tons of rusty waste. In 2013, the global cost of corrosion is nearly US $2.5 trillion [1]. The rust amount may be worth about 5% of the gross USA product. Consequently, the rusting of iron becomes a problem of worldwide significance. As a result, the Fe_2_O_3_ nanoparticles fabricated from rusted iron wastes can thus be considered as a viable alternative to synthetic and natural iron supplies. Many techniques have been used to prepare Fe_2_O_3_ nanostructures such as hydrothermal, chemical precipitation, sol-gel, thermal evaporation, and spray pyrolysis [2]. Most of these techniques needed high energy, complicated reactions, and poor product yield. Chemical precipitation is considered the most effective method for the production of Fe_2_O_3_ because no special additives or equipment are required.

On the other side, dye pollutants are becoming a major source of environmental contaminations [3,4]. Methylene blue (MB) is amongst the most commonly used dyes in textile industries. It causes substantial harm to both marine and human health due to its extremely carcinogenic and mutagenic [5]. Being exposed to MB dye can cause increasing nausea, heart rates, diarrhea, shock, vomiting, stomach cramp, and human tissue necrosis [6]. Therefore, the development of effective pollutant disposal techniques from wastewater at a reasonable cost is important to many research areas. Organic dyes are removed via physical, chemical, and biological techniques. Some of the techniques utilized include adsorption, filtration, membrane separation, coagulation, dilution, flotation, softening electrochemical, reverse osmosis, and photodegradation [3,7]. The technique of photodegradation is commonly used to remediate organic dyes from wastewater into harmless inorganic molecules such as CO_2_ and H_2_O [7,8].

Fe_2_O_3_ is widely used as a photocatalyst for the degradation of a wide range of pollutants due to its high chemical stability, low cost, and abundance [9,10]. Fe_2_O_3_ is a semiconductor material with a narrow bandgap. So, it has good light absorption in the visible region. However, this material has several drawbacks that limit its usage in photocatalytic applications, including quick electron-hole recombination, weak conductivity, and short hole diffusion lengths [11]. To overcome these drawbacks, several studies immobilized the Fe_2_O_3_ nanoparticles on various supports such as silica, zeolite, clay, and activated carbon. Among these, zeolite is a very fascinating material for environmental applications. Zeolite is a microporous aluminosilicates mineral based on TO_4_ in a tetrahedral structure, where; T is silicon or aluminum atoms [12]. In addition to its semiconducting nature, it has a strong adsorption ability for organic dyes. Zeolite has excellent ionic exchange properties with a high surface area, which makes it ideal for the degradation/adsorption of organic impurities. In addition, zeolite is biocompatible, abundant, and inexpensive.

Various types of zeolites supported photocatalysts have been investigated in the past few years. Mostafa and Ehab prepared zeolite nanostructures by the hydrothermal treatments of Si and various Al sources for the adsorption and photocatalytic degradation of MB molecules from aqueous media [13]. Zhang et al. prepared TiO_2_-zeolite photocatalysts through a hydrolysis deposition method combined with a calcination crystallization process [14]. Owing to improved adsorption of Rhodamine B (RhB) dye with effective delocalization of photogenerated electrons, the presence of zeolites increased the photocatalytic efficiency of the TiO_2_. [15]. This photocatalyst displayed superior photocatalytic performance with high stability and reusability. Alamri et al. prepared Fe_2_O_3_-supported zeolite for the removal of the ofloxacin [16]. The dealumination of zeolite accompanied by ion exchange of Al with Fe^3+^ in the zeolite framework resulted in the formation of active species that improved the photocatalytic activity toward the discoloration of MO [16]. Abukhadra et al. synthesized photocatalyst of zeolite/polyaniline/nickel oxide to effectively photodegrade the Safranin-T dye using sunlight [17]. WO_3_ and magnetite (Fe_3_O_4_) based zeolite composite was applied for photocatalytic degradation of rhodamine B dye using solar light [18]. The acceleration of RhB’s degradation rate was due to the efficient separation of the electrons/holes and the availability of active sites to adsorb RhB molecules [19].

This study aims to replace the iron precursors with rust wastes as a source of iron for the synthesis of Fe_2_O_3_ and Fe_2_O_3_-zeolite by low-cost chemical precipitation. The prepared Fe_2_O_3_-zeolite photocatalyst is applied for the photodegradation of the harmful MB dye. By this strategy, two issues related to water pollution and waste management can be solved simultaneously. According to the best of our knowledge, no articles are available on the recycling of the rusted iron wires for the fabrication of Fe_2_O_3_ and its coupling with zeolite for photodegradation. The prepared Fe_2_O_3_-zeolite photocatalyst shows the high photocatalytic performance comparing with the previous studies for Fe_2_O_3_-based. The photocatalytic performance is evaluated in terms of catalyst mass, starting dye concentration, exposure time, and pH value. Moreover, the stability, reusability, and photocatalytic mechanism of the Fe_2_O_3_-zeolite photocatalyst are investigated.

## 2. Materials and Experimental Procedures

### 2.1. Materials

Natural zeolite was delivered from a zeolite mine located in Taiz city, Yemen. Rusted iron wires were collected from construction sites. Methylene blue dye and sodium hydroxide were received from El-Nasr Company (Cairo, Egypt). Ammonia, hydrochloric acid, and hydrogen peroxide solutions were purchased from Rankem Company (New Delhi, India).

### 2.2. Preparation of the Fe_2_O_3_ and Fe_2_O_3_-Zeolite

For the preparation of the Fe_2_O_3_, small rusted wires were washed several times using distilled water. About 5 g of these pieces were dissolved in 40 mL of HCl, 20 mL of H_2_O_2_, and 80 mL distilled water for 3 h at 80 °C. At this stage, the Fe^3+^ and Fe^2+^ ions were obtained in the HCl solution. Under intense stirring, about 15 mL of ammonia solution with concentration of 25 wt% was dropped to the iron solution. The pale-yellow colored solution has appeared. After that, an appropriate amount of NH_3_ was added (1:1 with water) to obtain a dark brown precipitate of iron oxide hydroxide (FeOOH). The precipitate was washed several times via a centrifugation process operating for 10 min. Then, the resulting precipitated (FeOOH) powder was washed and dried in the air at 80 °C. Finally, FeOOH was calcined at 550 °C to obtain hematite nanoparticles (α-Fe_2_O_3_ NPs). On heating, the FeOOH loses a water molecule to form α-Fe_2_O_3_ according to the chemical reaction: FeOOH → Fe_2_O_3_ + H_2_O.

For the preparation of Fe_2_O_3_-zeolite photocatalyst, 5 g of raw zeolite was triggered mechanically by ball milling at 5000 rpm for 8 h. Then, it was washed with distilled water and dried in the air. About 1 g of Fe_2_O_3_ and 1 g of activated zeolite were added to 100 mL of distilled water for 2 h under ultrasonication. The resulting mixture was dried at 70 °C for 10 h. Finally, the Fe_2_O_3_ and Fe_2_O_3_-zeolite photocatalysts were calcined at 550 °C for 2 h. 

### 2.3. Characterizations

The X-ray diffraction (XRD) analysis is carried out by a XRD diffractometer (Philips X’Pert Pro MRD, Almelo, Netherlands) with λ = 0.154 nm and an operating voltage of 40 kV for obtaining the crystal structure of the zeolite, Fe_2_O_3_, and Fe_2_O_3_-zeolite. The samples morphologies were observed using a scanning electron microscope (SEM, JEOL JSM-5400 LV, Tokyo, Japan). The chemical composition was examined using energy-dispersive X-ray spectroscopy (EDX, JEOL JED-2300T, Tokyo, Japan). The optical properties of the samples were measured by UV-Vis double beam spectrophotometer (Perkin-Elmer Lamba 900, Waltham, MA, USA). Fourier transform-infrared (FTIR) spectra of photocatalysts were examined through FTIR spectrometer (Bruker Vertex 70 FTIR-FT, Billerica, MA, USA).

### 2.4. Photocatalytic Removal of MB Dye

The photocatalytic properties of Fe_2_O_3_-zeolite photocatalyst were examined toward MB dye as a typical organic pollutant under sunlight illumination. The photocatalytic measurements were performed in a cone-shaped vessel under stirring (500 rpm) at Beni-Suef city (Egypt) on sunshiny winter days (February 2021) from 10 am to 3 pm. The sunshine angle is 104^0^ east-southeast and has an average temperature of ~20 °C. Figure S1 shows a schematic diagram for the experimental photocatalytic measurements. Once the adsorption/desorption equilibrium was reached after 30 min, the photodegradation performance was examined versus the exposure times, catalyst masses, starting MB concentrations, pH values, and the number of reusability runs of the photocatalyst. The MB dye samples were taken each 10 s to observe using the Perkin-Elmer spectrophotometer at λ = 664 nm after regular exposure times.

#### 2.4.1. Influence of Exposure Time and Catalyst Supporting Role

The influence of exposure period on the photocatalytic removal of MB was studied using 20 mg and 60 mg of the targeted photocatalyst (zeolite, Fe_2_O_3_, or Fe_2_O_3_-zeolite) in 10 mg/L dye solution (100 mL) at pH 7 and 20 °C. Zeolite’s supporting role in improving the catalytic performance of Fe_2_O_3_ was studied by applying the Fe_2_O_3_ and Fe_2_O_3_-zeolite for the catalytic photodegradation of MB based on a comparative analysis.

#### 2.4.2. Influence of Starting MB Concentration

The influences of the starting MB concentrations (5–30 mg/L) on the MB dye removal-time characteristics were studied. This study was carried out using 20 mg of Fe_2_O_3_-zeolite catalyst in 100 mL MB dye solutions at pH 7 and 20 °C.

#### 2.4.3. Influence of the Photocatalyst Dose

Effects of the Fe_2_O_3_-zeolite photocatalyst dose (20, 35, 50, 62, and 75 mg) on the photodegradation of MB dye were tested up to 30 s exposure time. The measurements were carried out using 10 mg/L MB dye solutions at pH 7 and 20 °C. In addition, the average MB dye removal% as a single point measurement at 30 s using different catalyst masses are measured using 10 mg/L MB dye solutions at pH 7 and 20 °C.

#### 2.4.4. pH-Value Influence

0.1 M NaOH and HCl solutions are employed for controlling the pH-value of 100 mL MB solutions of starting 10 mg/L dye concentration. The influences of the pH-values on the photodegradation of the prepared MB solutions were studied using 20 mg of the photocatalyst under exposure to the natural sunlight for 30 s at ~20 °C.

#### 2.4.5. Stability of the Fe_2_O_3_-Zeolite Photocatalyst

The reusability of the Fe_2_O_3_-zeolite photocatalyst for MB dye degradation was studied for 10 successive runs. The measurements were carried out for 30 s using 75 mg of the Fe_2_O_3_-zeolite and 10 mg/L starting MB at pH 7. Before each re-usability, a washing process was performed with H_2_O and a drying process at 70 °C for 120 min. In this case, 10 cycles of reusability were performed using sunlight every 30 s.

#### 2.4.6. Chemical Oxygen Demand (COD) Measurements

The sample was oxidized by a boiling mixture of chromic and sulfuric acids and refluxed for 2h in a strong acid solution with a known excess of potassium dichromate. After digestion, the remaining unreacted K_2_Cr_2_O_7_ was titrated with ferrous ammonium sulfate to determine the amount of K_2_Cr_2_O_7_ consumed. The oxidizable matter was calculated in terms of oxygen equivalent. The measurements were carried out in triplicates in order to acquire accurate data.

## 3. Results and Discussion

### 3.1. Characterization of the Photocatalysts

#### 3.1.1. Surface Morphology

**Figure 1:**
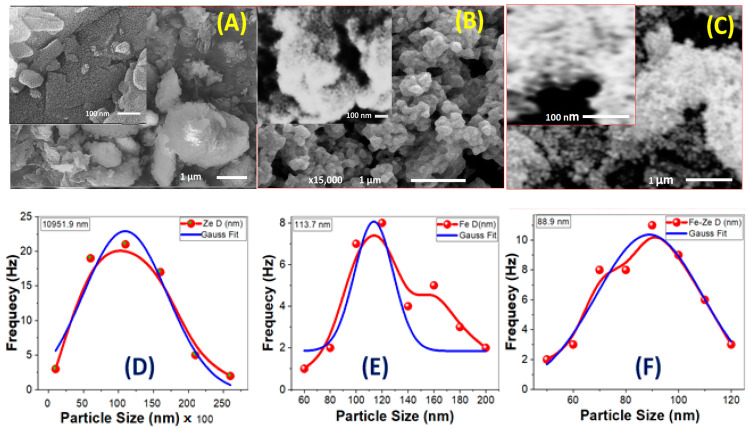
SEM micrographs and the corresponding particle size distribution for (**A**,**D**) natural zeolite, (**B**,**E**) Fe_2_O_3_, and (**C**,**F**) Fe_2_O_3_-zeolite.

The photocatalytic activity of a photocatalyst is widely recognized to be substantially related to its surface shape. Figure 1A–C depicts the SEM examination of the morphologies of natural zeolite, Fe_2_O_3_, and Fe_2_O_3_-zeolite nanopowders.

Figure 1A shows SEM pictures of natural zeolite with micro/nano-stones in nonuniform shapes and sizes. The diameters of the stones for zeolite are varied from ~3.0 to ~26 μm, as evidenced in the matching particle size distribution (Figure 1D). Using Gaussian fitting, the mean stone size is ~11 μm with a standard deviation of 6.0 μm. A detailed examination of the image indicates the presence of numerous tiny nanoprotrusions/nanograins on the surfaces of zeolite stones with an average size of 115 nm. In addition, as observed in high magnification Figure 1A, there are many macropores among agglomerated zeolite crystals with an average diameter of ~70 nm on the surface of zeolite with irregular forms. The porous framework’s high surface area allows for the incorporation of iron oxide nanoclusters on the surface of the zeolite. These pores can also adsorb organic contaminants, which can improve photodegradation efficiency.

The Fe_2_O_3_ nanoparticle was made up of numerous semi-spherical nanoparticles. Figure 1B displays SEM images of Fe_2_O_3_ nanoparticles that demonstrate the nanoparticles are modest in size. Figure 1E shows the appropriate particle size distribution. The mean size of Fe_2_O_3_ nanoparticles is ~114 nm, with a standard deviation of ~15 nm, according to Gaussian fitting. These nanoparticles self-assemble and aggregate to create mesopores with an average diameter of ~21 nm and a standard variation of ~6 nm, as illustrated in Figure 1B’s inset.

Fine spherical Fe_2_O_3_ nanoparticles coated the zeolite surface and appeared as homogenous distributions that produced a nano-sized Fe_2_O_3_ coating surface over zeolite stones after loading zeolite with the intended Fe_2_O_3_ photocatalyst, as shown in Figure 1C. It’s also possible that the Fe_2_O_3_ coating was relatively uniform, with no obvious areas of uncoated zeolite. When compared to free-standing Fe_2_O_3_ nanoparticles, the size of the Fe_2_O_3_ nanoparticles appears to be reduced following loading on zeolite. The size distribution of supported Fe_2_O_3_ nanoparticles on the surface of zeolite, Figure 1F, shows an average value of ~89 nm. In addition, the high magnification SEM picture (inset of Figure 1C) reveals a more homogeneous pore-diameter distribution with a mean value of ~36 nm.

The interlock of Fe_2_O_3_ photocatalytic activity. Tedla et al. demonstrated that comparable interlock arrangements can allow generated electrons and holes to migrate quickly to the catalyst surface, resulting in a low chance of recombination [20]. Furthermore, reducing the particle size to the nanoscale and increasing the pores can provide a large effective surface area of the Fe_2_O_3_ nanocatalyst. This can boost the catalyst’s adsorption capability and allow for more intense absorption of incident light.

#### 3.1.2. Chemical Compositions of the Photocatalysts

The EDX spectra were presented in Figure S2 (supplementary data) to identify the chemical compositions of zeolite, Fe_2_O_3_, and Fe_2_O_3_-zeolite photocatalysts. As seen in Figure S2a (supplementary data), the chemical composition for the zeolite displays the main three elements (Si, Al, and O). Smaller traces of Fe, Cu, K, and Ca have also been observed [21]. From Figure S2b (supplementary data), the EDX analysis of Fe_2_O_3_ showed the existence of Fe (62.3%) and O (37.6%) signals as the main components at around 6.39 and 0.52 keV, respectively. After loading Fe_2_O_3_ onto zeolite, the main characteristic peaks are Fe, Si, Al, and O with atomic ratios of 8.9%, 23.6%, 6.3%, and 58.0%, respectively, as seen in Table 1.

Zeolite is divided into heulandite and clinoptilolite which are isostructural with each other. Heulandite and clinoptilolite were differentiated by their framework composition. The Si/Al ratio is a critical value to distinguish between heulandite and clinoptilolite. Heulandite has Si/Al ratios between 2.8 and 4. Clinoptilolite has a Si/Al ratio > 4. Heulandite is encountered containing Ba, Ca, Na, K, and Sr. The ratio of atomic Si/Al ratios is increased from 3.61 for zeolite to 3.74 for zeolite-Fe_2_O_3_. Hence, the Si/Al is smaller than 4 for zeolite and Fe_2_O_3_-zeolite. Therefore, the type of zeolite (heulandite) not changes after adding Fe_2_O_3_. This suggests that Fe_2_O_3_ was successively loaded onto the porous surface of the zeolite.

#### 3.1.3. Structural Properties of Fe_2_O_3_ and Fe_2_O_3_-Zeolite Photocatalyst

**Figure 2:**
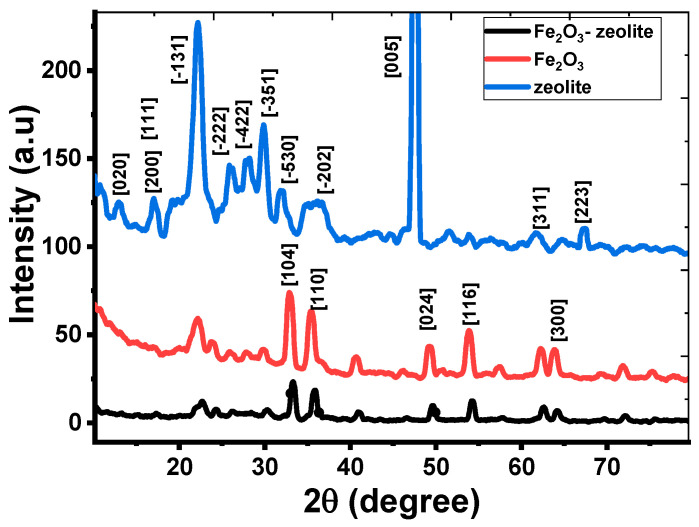
XRD patterns of zeolite, Fe_2_O_3_, and Fe_2_O_3_-zeolite photocatalysts.

The phase and crystallinity of the zeolite, Fe_2_O_3_, and Fe_2_O_3_-zeolite photocatalysts were studied by using XRD analysis as displayed in Figure 2.

Based on XRD data, zeolite has a tetragonal structure according to JCPDS No. 00-053-1176. The type of zeolite is heulandite-Ca with the chemical formula Al_8.8_Ca_3.6_H_52.2_K_0.8_O_98.1_Si_27.4_. The heulandite-Ca is hydrous calcium and aluminum silicate. A small amount of potassium is usually replaced with part of the calcium. It has XRD peaks at 2θ = 11.00, 17.16, 18.87, 22.24, 26.01, 27.97, 29.84, 31.83, 35.88, 47.58, 61.76, and 67.31°. These peaks match to the planes [020], [200], [111], [-131], [-222], [-422], [-351], [-530], [−202], [005], [311] and [223], respectively.

The XRD pattern in Figure 2 indicates that crystalline hematite (α-Fe_2_O_3_) with rhombohedral structure was created for iron oxide nanoparticles based on JCPDS No. 01-089-0597. The peaks of Fe_2_O_3_ are located at 2θ = 33.00, 35.39, 49.32, 53.84, and 63.74°, which, respectively, relate to the planes [104], [110], [024], [116], and [300]. The sharp peaks suggest the high crystallinity of the synthesized hematite nanoparticles using the rusted iron wires. This indicates the high purity of the prepared Fe_2_O_3_ nanostructure, which agrees with the EDX results. According to the Debye-Scherrer relation, the crystallite sizes of Fe_2_O_3_ nanoparticles for [110] and [104] are 50.46 and 64.84 nm, respectively.

Figure 2 clarified that the primary core characteristics of Fe_2_O_3_ and Fe_2_O_3_-zeolite charts are highly similar, which showing that the addition of zeolite did not affect the crystal structure of the Fe_2_O_3_ photocatalyst. The coupling of zeolite with Fe_2_O_3_ results in an increase in the FWHM and a move in the plane location of the Fe_2_O_3_ toward higher angles for the Fe_2_O_3_-zeolite photocatalyst. Therefore, the crystallites sizes of [110] and [104] peaks were reduced to 47.85 and 56.53 nm for Fe_2_O_3_-zeolite photocatalyst, respectively. The same behavior was observed for zeolite-supported Fe_2_O_3_ prepared by the hydrothermal method [22]. Furthermore, the intensities of the diffraction peaks of Fe_2_O_3_-zeolite photocatalyst became lower than those of Fe_2_O_3_, representing a change in the crystallinity due to the distribution of Fe_2_O_3_ on the surface of the zeolite [23].

The structural parameters of Fe_2_O_3_ and Fe_2_O_3_-zeolite photocatalysts such as crystallite size (d), dislocation density (δ), interplanar distance (d), and microstrain (ε) are calculated for the highest two peaks, [110], [104] and listed in Table S1 (supplementary data). After loading the zeolite with Fe_2_O_3_, the value of interplanar distance decreases while the microstrain increases for the two planes [104] and [110]. The strongest peak corresponds to the plane [104], which indicates the preferred growth orientation of hematite. This growth orientation is beneficial to carrier transport [24]. The number of lattice defects was estimated depending on the dislocation density (δ). The value of δ is calculated by using the relation; δ = D ^−^^2^. The values of δ at the orientation [104] for the Fe_2_O_3_-zeolite and Fe_2_O_3_ are 3.129 × 10^−4^ and 2.378 × 10^−4^ nm^−^^2^, respectively. The high density of the defects might contribute favorably to the photocatalytic performance of the Fe_2_O_3_-zeolite photocatalysts as a result of the formation of a high density of the active sites and the surface area increase [25]. The creation of static charge around the dislocation lines may result in the formation of these active sites.

#### 3.1.4. Functional Groups

**Figure 3:**
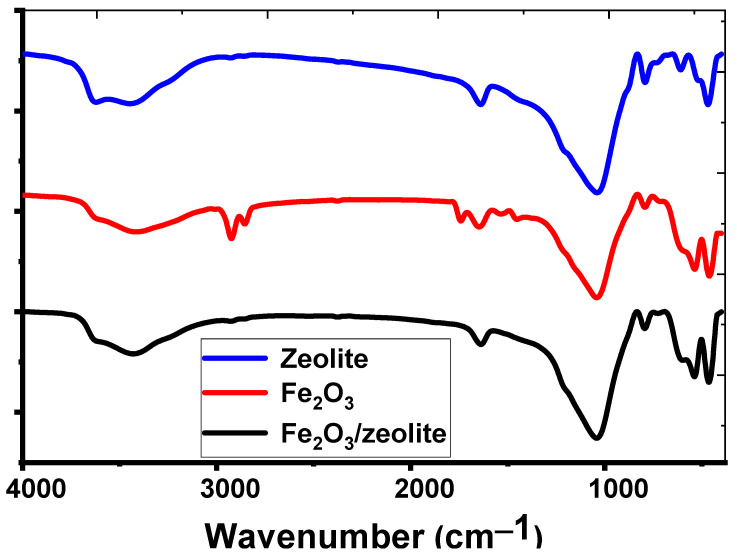
FTIR spectra of zeolite, Fe_2_O_3_, and Fe_2_O_3_-zeolite photocatalyst.

Figure 3 illustrates a comparison of the different photocatalysts’ FTIR spectra ranges. Figure 3 shows the FTIR spectra of Fe_2_O_3_ nanoparticles in the 4000–400 cm^−^^1^ wavenumber band. There are absorption bands at 1641 and 3415 cm^−^^1^ in the case of Fe_2_O_3_. The absorbed H_2_O bending vibrations, O-H stretching vibrations, and surface hydroxyl modes were all attributed to these bands. Due to the symmetric and asymmetric -CH_2_-groups stretch modes, there are some absorption bands at 2920 and 2850 cm^−^^1^. These groups may be due to hydrocarbon contamination in the surface of rusted iron wire. At 1040 cm^−^^1^, a sharp absorption peak was seen, which was attributed to the Fe-O asymmetric stretching mode [26]. The FTIR spectrum of zeolite, on the other hand, was distinct, as illustrated in Figure 3. Si-OH groups with H-bonding were found to have broadbands at 3620 and 3446 cm^−^^1^. The OH bending mode is responsible for an absorption peak at 1640 cm^−^^1^ [27]. The O–Si(Al)–O bond vibrations in tetrahedra (TO_4_) or aluminum- and silicon-oxygen bridges are responsible for the intensive band at 1040 cm^−^^1^ [27]. In the region of 700–500 cm^–1^, the bands were ascribed to four- or five-membered ring vibrations of TO_4_ units and stretching of the inter tetrahedral bonds typical of the ordered crystal structure, respectively [27].

Figure 3 shows the FTIR of Fe_2_O_3_-zeolite. Broadband was discovered at 3429 cm^−^^1^ and attributed to the O-H stretch mode, whereas the O-H bending mode was observed at 1650 cm^−^^1^ [28]. At 1000 cm^−^^1^, zeolite bands appear in the photocatalyst, and the shift of these bands relative to zeolite refers to H-bond breaking produced by Fe on zeolite SiO_4_/AlO_4_ surfaces. Strong bands at 720, 598, 530, and 460 cm^−^^1^ were attributed to the symmetric vibration of (Al or Si)-O due to the internal vibration of zeolite.

#### 3.1.5. The Photocatalysts’ Optical Properties

**Figure 4:**
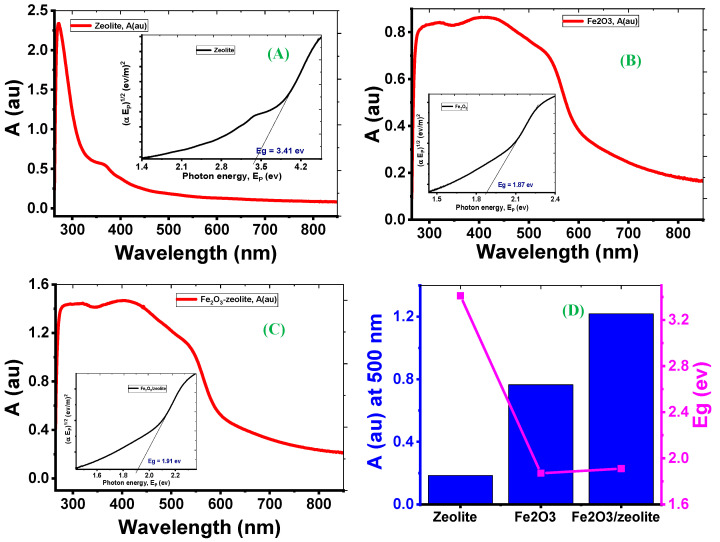
Absorbance and Tauc curve for zeolite (**A**), Fe_2_O_3_ (**B**), and Fe_2_O_3_-zeolite (**C**); and absorbance at 500 nm and indirect Eg for all samples (**D**).

Figure 4 shows the absorption spectra of zeolite, Fe_2_O_3_, and Fe_2_O_3_-zeolite. The zeolite powder presents a prominent peak due to a substantial photoabsorption band in the ultraviolet (UV) region, as seen in Figure 4A. As the wavelength becomes longer, the absorption drops rapidly. This indicates that the zeolite sample’s spectral response in the visible light region was exceedingly low. Figure 4B,C shows that the optical absorbance of Fe_2_O_3_ was modified after loading Fe_2_O_3_ onto zeolite. The Fe_2_O_3_ has strong absorption in the visible and UV regions [29]. Due to the spin-forbidden-excitations and straight transition, Fe_2_O_3_ has a broad absorption band in the visible region [30,31,32]. A wide and sharp absorption band in the visible region was detected for the Fe_2_O_3_-zeolite as seen in Figure 4C. The absorbance values of zeolite, Fe_2_O_3_, and Fe_2_O_3_-zeolite photocatalysts are 0.18, 0.76 and 1.22 at λ = 500 nm, respectively, Figure 4D. This means more and more photons in the visible region can be absorbed by Fe_2_O_3_-zeolite than Fe_2_O_3_. This would be preferable to generate a large number of electron-hole pairs by transferring electrons from the valence to conduction bands. As result, zeolite has significantly improved the visible light absorption capability of the loaded Fe_2_O_3_ photocatalyst.

The indirect band gaps of Fe_2_O_3_, zeolite, and Fe_2_O_3_-zeolite photocatalysts were expected by Tauc’s equation ${\left({\alpha \;E}_{p}\right)}^{0.5}=\alpha_{0}\;\left(E_{p}-E_{g}\right)$. Where $E_{p}$ is the energy of the incident photon light, $E_{g}$ refers to the optical bandgap, α is the absorption coefficient, and $\alpha_{0}$ represents independent energy constant. This demonstrates the formation and incorporation of Fe_2_O_3_ nanoparticles in the zeolite. These results agree with the stated values for Fe_2_O_3_ fabricated by many methods [16,33]. Based on the quantization effect, the decrease in crystallite size reported in XRD data can explain the rise in the bandgap of Fe_2_O_3_-zeolite compared to Fe_2_O_3_ [34,35]. The studied optical properties suggest that Fe_2_O_3_-zeolite can be used for solar energy applications such as the photodegradation of organic dyes under sunlight.

### 3.2. Evaluation of Photocatalytic Performance

#### 3.2.1. Role of the Catalyst Support and Effect of Irradiation Time

**Figure 5:**
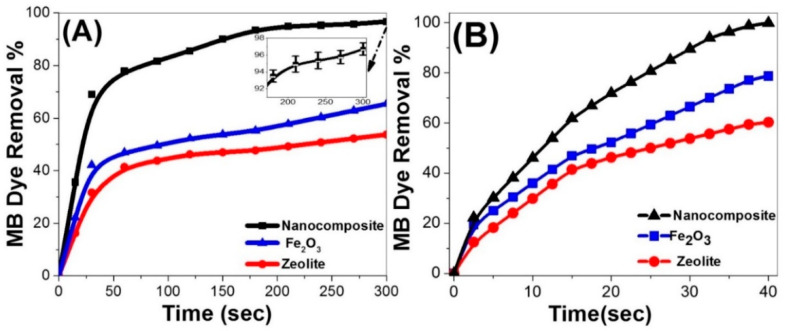
The photocatalytic degradation of 100 mL MB dye solution of concentration 10 mg/L at pH 7 using zeolite, Fe_2_O_3_, and Fe_2_O_3_-zeolite photocatalyst (**A**) 20 mg, and (**B**) 60 mg. The inset shows the statistical error bars.

The photocatalysis tests were performed over Fe_2_O_3_, zeolite, and Fe_2_O_3_-zeolite to recognize their influences on the photodegradation performance and to evaluate the role of zeolite for enhancing the photocatalytic behavior of Fe_2_O_3_ for MB photodegradation utilizing the sunlight (Figure 5). The values of the photocatalytic removal% within 300 s, Figure 5A, are 53%, 65%, and 96% for the photocatalytic degradation of MB using 20 mg of zeolite, Fe_2_O_3_, and Fe_2_O_3_-zeolite, respectively. With increasing the catalyst dose to 60 mg and 40 s exposure time, Figure 5B, the photocatalytic degradation efficiency is jumped to reach ~60%, 78%, and 99% using zeolite, Fe_2_O_3_, and Fe_2_O_3_-zeolite, respectively. These results showed that the existence of zeolite supporting can play the main role in improving the photodegradation rate of the photocatalyst. This improvement is attributed to two factors. First, zeolite is considered an electron acceptor and contributes effectively to the enhancement of the electron transfer from the photoexcited Fe_2_O_3_ to the host zeolite molecules. Consequently, it prevents electron-hole recombination [36]. Second, it reflects several beneficial attributes, such as its high effective surface areas and strong adsorption property.

From Figure 5, MB dye removal% increases continuously with increasing the exposure time for all catalysts until a specific time and then reaches a plateau value. Comparing the time-scale photodegradation profiles of MB over zeolite, Fe_2_O_3_, and Fe_2_O_3_-zeolite under sunlight illumination, an efficient photodegradation of MB is observable utilizing 60 mg of Fe_2_O_3_-zeolite within 40 s. The high performance of the Fe_2_O_3_-zeolite photocatalyst is due to its ability to grow charging carriers formed by incident photons in the development of oxidizing radicals such as hydroxyl radicals/superoxide anions. The oxidizing radicals density in the photocatalytic cell controls the photocatalyst’s effectiveness in moving the charge carriers and degrading the organic pollutants.

#### 3.2.2. Influence of Starting Dye Concentrations and Photocatalyst Dosage

**Figure 6:**
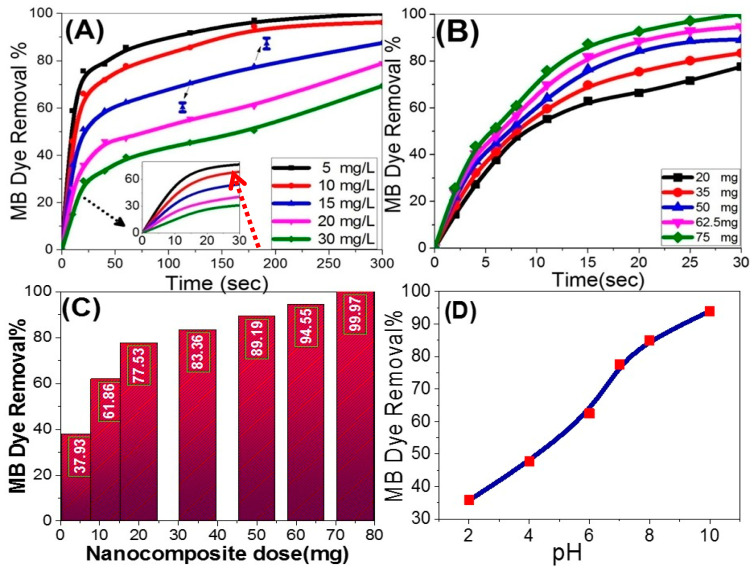
Effects of (**A**) initial dye concentration over 20 mg Fe_2_O_3_-zeolite and (**B**) photocatalyst dose for 10 mg/L MB on the time-scale photodegradation profiles of MB; MB dye removal as a function of (**C**) photocatalyst dose and (**D**) pH value using 20 mg of the catalyst over 100 mL MB under 30 s sunlight illumination at 20 °C.

The influence of the starting dye concentrations (5–30 mg/L) on the time-scale photodegradation characteristics of 100 mL MB over 20 mg of Fe_2_O_3_-zeolite under sunlight illumination was studied at pH 7 and 20 °C. The graphical representation of these time-scale photodegradation profiles is shown in Figure 6A. As the exposure time increased to 300 s, the photocatalytic MB dye removal% increases to reach 100, 96, 87, 78, and 69% at starting dye concentrations of 5, 10, 15, 20, and 30 mg/L, correspondingly. In this case, i.e., the MB photodegradation rate is decreased by rising the starting MB concentration. As shown in the inset of Figure 6A, the plateau removal% increased and reached after a short time by decreasing the initial dye concentration. This may be because the dye molecules at high concentrations serve as an optical filter to the incident light, so that, this may be because the dye molecules at high concentrations serve as an optical filter to the incident light, so that the desired light intensity may not enter the photocatalyst [37]. This is also due to the scarcity of the lifetime of the produced hydroxyl radicals (just a few nanoseconds), they can only react at or near the place where they are produced [37].

The determination of the optimum dose of photocatalysts is a key point in the scaling up of the catalytic method since it touches the financial side and separation process of the catalyst from the MB solution. The effects of the Fe_2_O_3_-zeolite dose (20, 35, 50, 62, and 75 mg) on the time-scale photodegradation curves of 100 mL MB (10 mg/L) were tested up to 30 s exposure time at pH 7 and 20 °C, Figure 6B. As the exposure time increased, the MB dye removal% increased to reach 77% over 20 mg Fe_2_O_3_-zeolite after 30 s. The complete removal of the MB dye is reached at 30 s by increasing the dose of Fe_2_O_3_-zeolite to 75 mg. Increasing the catalyst dose speeds the photodegradation cycle by supplying more active sites to generate free radicals [38,39]. In addition, the average MB dye removal% of triplicate measurements are presented as a single point data in Figure 6C at 30 s using different catalyst masses over 10 mg/L MB dye solutions at pH 7 and 20 °C. The values of the average removal% were 37, 61, 77, 83, 89, 94, and 99% by using 5, 12.5, 20, 35, 50, 62.5, and 75 mg of Fe_2_O_3_-zeolite photocatalyst, respectively. Then, the amount of applied Fe_2_O_3_-zeolite to the photocatalytic system has a major impact on process performance. In this case, i.e., the photocatalytic performance improvement is correlated with an increase in the heterogeneous catalyst dose. This can be understood based on the improvement in the catalyst’s surface area and the availability of more dynamic sites for the discoloration of dye solution [40,41].

#### 3.2.3. Influence of pH Value

The optimized pH value is a critical characteristic that directly influences the organic pollutants and photocatalyst’s net surface charges [42]. The influence of pH value on the photocatalytic removal% of MB was illustrated in Figure 6D. These measurements were carried out using 20 mg of Fe_2_O_3_-zeolite over 100 mL MB (10 mg/L) under 30 s sunlight illumination at 20 °C. There are noticeable increases in MB removal%, as the pH rises from 2 to 10. The removal% increased from 35 to 93% as the pH increased from 2 to 10. The efficient MB photodegradation was observed at high pH values (alkaline solutions) due to the surface deprotonation. Therefore, the photocatalyst surfaces are negatively charged which improves the MB (basic dye) uptake [43].

#### 3.2.4. Reusability of the Fe_2_O_3_-Zeolite Photocatalyst

**Figure 7:**
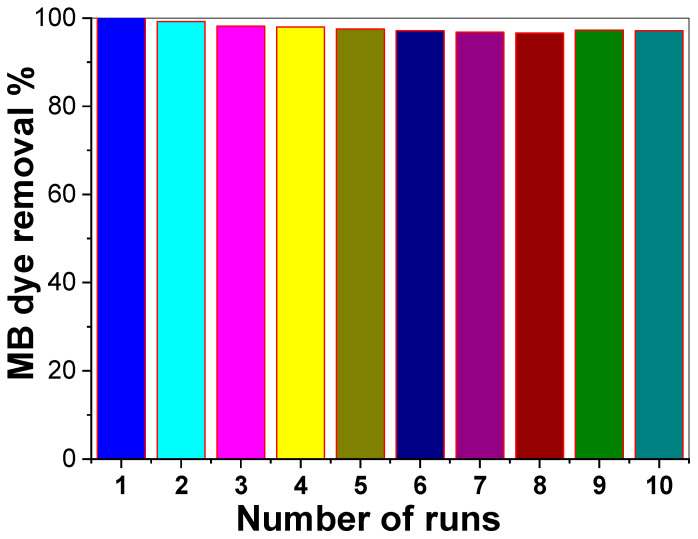
The reusability of 75 mg of Fe_2_O_3_-zeolite photocatalyst for degradation 10 mg/L of MB for 10 runs at pH 7 for 30 s.

Reusability/stability is an important feature of the functional application of the Fe_2_O_3_-zeolite catalyst. The photocatalytic stability and reusability of Fe_2_O_3_-zeolite in the photodegradation of MB dye were subjected to 10 successive cycles of reusability (Figure 7) using 75 mg from the photocatalyst with 100 mL of 10 mg/L MB within 30 s under sunlight. The recovery process after each run was performed by washing and drying the used photocatalyst at 70 °C for 2 h. The catalytic photoreactivity of the Fe_2_O_3_-zeolite displays a little loss which revealed the high photostability of the catalyst upon exposure to sunlight radiation. In addition, the photodegradation percentage of dye using Fe_2_O_3_-zeolite photocatalyst displays high performance for the 10 cycles investigated at 30 s each. After the tenth run, the photocatalytic activity of the Fe_2_O_3_-zeolite was reduced from ~100 to 9%. So, after 10 recycling cycles, the Fe_2_O_3_-zeolite can retain ~97% of its initial performance. This confirms that Fe_2_O_3_-zeolite nanocatalyst is extremely stable and recyclable.

#### 3.2.5. Kinetics of Photocatalytic Process

Facilitating the wide-ranging application of the photocatalyst requires understanding and exploring the photocatalytic reaction kinetics. The MB concentration-time curves were used to identify the suitable catalytic photodegradation kinetics for MB. In the heterogeneous photocatalytic process, the photocatalyst cleaves the aromatic ring of the dye molecules and initiates its degradation. The kinetic modeling explains the nature of the photocatalytic mechanism. The kinetic models’ parameters, containing the photocatalytic degradation rates, have been calculated by presented C_t_, ln(C_0_/C_t_), and 1/C_t_ vs. exposure time (t) for zero-order, pseudo-first-order, and pseudo-second-order, correspondingly. In that order, the kinetic models are presented in Equations (1)–(3) [44].

C_t_ = C_0_ − k_0_ t (1)

C_t_ = C_0_ e^−(k_1_^^t)^ (2)

1/C_t_ = 1/C_0_ + k_2_ t (3)

where, k_n_ refers to the resultant degradation rate (n = 0, 1, 2), and C_t_ represents reminder MB concentration after exposure time t.

**Figure 8:**
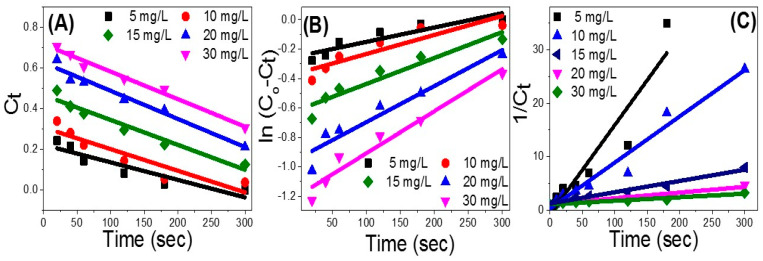
Photodegradation data fitting using (**A**) zero-order (**B**) first-order, and (**C**) second-order kinetic models.

The experimental curves were fitted based on Equation (1), zero-order-model, by plotting the residual dye concentration linear regression versus the illumination time (Figure 8A). For this model, the determination coefficient (R^2^) value displays a good fit, especially for dye concentration ≥ 15 mg/L, Table 2. In addition, the predicted photodegradation rate (k_0_) increases by rising the MB concentration from 5 to 20 mg/L and then remains constant at 14 × 10^−^^4^ min^−^^1^ until 30 mg/L (Table 2).

The plot of Ln(C_0_ − C_t_) vs. the exposure time, Figure 8B, was fitted based on Equation (2) for the first-order model. The obtained parameters in Table 2 show a moderate fit between the experimental results and this model for MB concentration < 15 mg/L, and then a better fit for dye concentrations > 20 mg/L. The photodegradation rate constant also displays a significant rise in the calculated values to reach 29 × 10^−^^4^ min^−^^1^, with rising the MB concentration to 30 mg/L.

The linear regression plotting of 1/C_t_ versus illumination time, by the same fitting process, reflects the fit of the photodegradation data to the kinetic second-order model (Figure 8C). The model’s output is displayed in Table 2. MB dye degradation tends to be matched to the second-order model at starting MB concentration of 10 mg/L and 15 mg/L. Then, the matching degree decreases for dye concentration > 20 mg/L. The value of k_2_ also displayed a steady decrease with rising the starting MB concentration consistent with the experimental data. The value of k_2_ is decreased from 1681 × 10^−^^4^ to 65.5 × 10^−^^4^ L/mol.min^−^^1^, with rising the starting MB concentration from 5 to 30 mg/L. Therefore, the experimental photodegradation results are well presented by the second-order model rather than any other model at starting MB concentration < 20 mg/L as shown in Table 2. Therefore, the photodegradation process is dye concentration- and catalyst dose-dependent. To identify the effect of catalyst dose in the photodegradation process similar fitting processes were carried out at different catalyst doses (20–75 mg) using Figure 6B and presented in Table 3. This table shows that the MB photodegradation follows pseudo-second-order kinetics.

For dye concentration ≥ 20 mg/L, the photodegradation process is well presented by the zero-order model instead of any other model, and the reaction rates are independent of the initial dye concentration. This is a common behavior that occurs when the reaction requires contact with a catalytic surface. The photodegradation data is fitted with more than one kinetic model to show concurrent and comparable degradation mechanisms for the studied starting MB concentrations [44]. It is important to note that the reported photodegradation rate constant, k_2_, is higher than any previously reported data for Fe_2_O_3_-based photocatalyst [24,45]. This can explain the fast photodegradation performance of Fe_2_O_3_-zeolite under visible light illumination.

Table 4 illustrates the photocatalytic performance of our Fe_2_O_3_-zeolite photocatalyst comparing with the previously studied metal oxide and/or zeolite-based photocatalysts for MB dye degradation [46,47,48,49,50,51,52,53]. As shown in this table, the presented photocatalytic performance parameters are much higher than the reported performances for the previously reported catalysts in the literature. As an example, the photodegradation efficiency of Ni-doped α-Fe_2_O_3_ photocatalyst was 89% after 140 min under visible light illumination [48]. In addition, Au-zeolites required 11 min under sunlight illumination to reach 50% dye removal [47]. Under the UV exposure, Fe_2_O_3_/Cu_2_O photocatalyst reached 91% dye removal% after 120 min, whereas, Fe_2_O_3_/graphene oxide required 100 min to reach the complete removal% [49,50].

#### 3.2.6. Chemical Oxygen Demand (COD) Measurements

The mineralization degree was estimated by analyzing the chemical oxygen demand (COD) in methylene dye solution under sunlight. This technique has been frequently used to quantify the organic waste water capacity. The COD of the dye solution was estimated before and after the treatment. The COD removal (%) was calculated from Equation (4);

(4) $$\mathrm{COD}\;\mathrm{removal}\;\left(\%\right)=\frac{\mathrm{Initial}\;\mathrm{COD}\;-\;\mathrm{Final}\;\mathrm{COD}\;}{\;\mathrm{Initial}\;\mathrm{COD}}\times 100$$

For aqueous solution of MB, the COD removal% was more than 91% after visible light irradiation for 60 min as seen in Figure S3 (supplementary data).

#### 3.2.7. Photocatalytic Mechanism of Fe_2_O_3_-Zeolite

**Figure 9:**
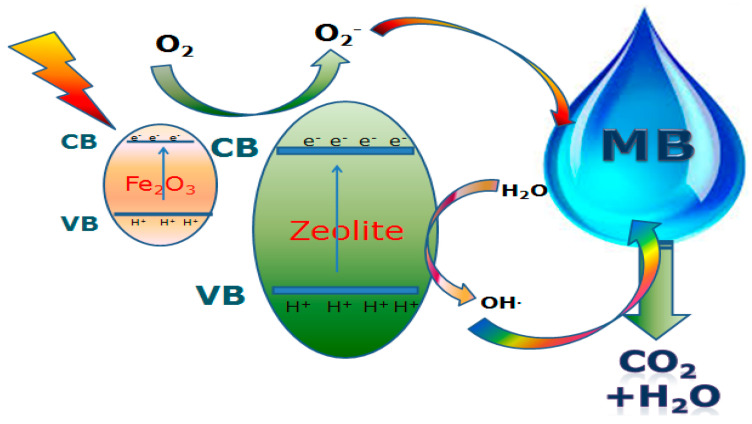
A schematic diagram of the proposed mechanism for MB photodegradation utilizing Fe_2_O_3_-zeolite under sunlight illumination.

Fe_2_O_3_ has been considered one of the main heterogeneous photocatalysts in the fields of solar energy conversion and environmental treatment, but it suffers from many disadvantages such as high load recombination, low conductivity, and short hole lengths in the bulk form [54]. The mesoporous surfaces of zeolite act as optimum sites to capture Fe_2_O_3_ nanoparticles. Adsorption is the starting stage in the photodegradation process before decoloration of MB molecules and is therefore a crucial mechanism for initiating the photodegradation process. Direct electrochemical bonding and electrostatic interactions are the principal photocatalytic reactions between photocatalyst surfaces and MB molecules. The electrochemical bonding between the functional dye groups and the active photocatalyst sites is a powerful interaction between the photocatalyst and the anchoring MB dye. Figure 9 shows a schematic diagram of the proposed mechanism of MB photodegradation using Fe_2_O_3_-zeolite under sunlight illumination. The mesoporous surfaces of zeolite serve as a large and strong holding place for Fe_2_O_3_ nanostructures and hot adsorption sites for the MB molecules at the start of the process. In addition, zeolite can distribute the exciting CB electrons of Fe_2_O_3_ into the network structure and thus reduce the recombination of electron-hole pairs [55]. Zeolite and Fe_2_O_3_ serve as active photoelectric sites during the photocatalytic cycle and require good interaction between the surfaces of MB and the photocatalyst for effective MB photodegradation. As shown in Figure 9, the Fe_2_O_3_ CB is marginally more negative whereas the VB is much more positive than the zeolite CB. It enhances the driving forces of the migration of holes relative to electrons migration. This causes a decrease of e^−^/h^+^ recombination rates and can thus result in an efficient charge carrier separation. Hence, the effective e^−^/h^+^ separation occurs over robust interfacial interactions in Fe_2_O_3_-zeolite heterostructure, which reduces the e^−^/h^+^ recombination and improves the photo/photoelectrocatalytic performance. In addition, the trapping of the electrons by the distributed Fe_2_O_3_ nanoparticles on the surface of the mesoporous zeolite can improve the photocatalytic performance of Fe_2_O_3_-zeolite. Whereas O_2_^•−^ (radical superoxide) is produced from the interaction between the trapped electron and dissolved oxygen in the photocatalytic system, which results in the development of hydroxyl radicals (•OH). The rate of oxidation of the organic compounds depends on the densities of these radicals. Additionally; the vigorous sites on the photocatalyst surfaces can play an important role in improving photocatalytic efficiency. Eventually, the cost of the engineered Fe_2_O_3_-zeolite photocatalyst is estimated to be ~0.5–0.7 USD per 1 g. However, for massive production, it can be reduced to 0.3–0.4 USD. Therefore, this study provided an innovative method for producing highly efficient photocatalysts for acceptable industrial applications at a reasonable cost.

## 4. Conclusions

In this paper, rusted iron wastes were recycling for the preparation of Fe_2_O_3_ and Fe_2_O_3_-zeolite photocatalyst. The use of zeolite as support has induced significant changes in the properties of Fe_2_O_3._ Compared to Fe_2_O_3_, the Fe_2_O_3_-zeolite photocatalyst had a more homogeneous mesopores diameter distribution, smaller sizes, and higher light absorption capabilities. Fe_2_O_3_-zeolite photocatalyst was applied successfully for the photodegradation of MB dye under sunlight illumination. It showed much higher photocatalytic performance against Fe_2_O_3_ nanopowder, which, to the best of our knowledge, is the highest performance yet for Fe_2_O_3_-based photocatalysts. Different photocatalytic parameters such as the exposure time, starting MB concentrations, photocatalyst dosage, and pH value have been optimized to reach the full photocatalytic degradation of MB dye (10 mg/L) within 30 s utilizing 75 mg of Fe_2_O_3_-zeolite. In addition, this photocatalyst showed extreme stability and recyclability because it can retain ~97% of its initial performance after 10 cycles of reusability. For initial dye concentration <20 mg/L, the photodegradation process followed the pseudo-second-order kinetics and showed a very high photodegradation rate constant. The present research can be applied industrially as a cost-effective technique to address two issues at once by quickly eliminating the harmful MB dye from wastewater and recycling the rusted iron wires.

## Figures and Tables

**Table 1:** The Atomic ratio of zeolite, Fe_2_O_3_, and Fe_2_O_3_-zeolite photocatalysts.

Element	Zeolite	Fe_2_O_3_	Fe_2_O_3_-Zeolite
O	65.2	37.6	58.0
Al	6.9	---	6.3
Si	25.0	---	23.6
K	1.3	---	1.4
Ca	0.9	---	1.1
Fe	1.6	62.4	8.9
Cu	0.1	---	0.7

**Table 2:** The zero-, first-, and second-order kinetic parameters using 20 mg Fe_2_O_3_-zeolite photocatalyst for photodegradation of MB of different concentrations.

Kinetic Model	Parameters	5 mg/L	10 mg/L	15 mg/L	20 mg/L	30 mg/L
Zero-order kinetic model	K_0_ (mol/(L·min)) R^2^	0.0009 ± 0.00018 0.8550	0.0011 ± 0.00022 0.8523	0.0012 ± 0.00014 0.9501	0.0014 ± 0.00012 0.9696	0.0014 ± 0.00009 0.9833
First-order kinetic model	k_1_ (/min) R^2^	0.0010 ± 0.00022 0.8329	0.001 ± 0.00030 0.8269	0.002 ± 0.00028 0.9085	0.002 ± 0.00035 0.9239	0.003 ± 0.00031 0.9553
Second-order kinetic model	k_2_ (L/mol min) R^2^	0.17 ± 0.027 0.9039	0.09 ± 0.007 0.9620	0.02 ± 0.002 0.9703	0.01 ± 0.001 0.9350	0.01 ± 0.0006 0.9456

**Table 3:** The zero-, first-, and second-order kinetic parameters using 10 mg/L MB for photodegradation of MB of different concentrations.

Kinetic Model	Parameters	20 mg	35 mg	50 mg	62.5 mg	75 mg
Zero-order kinetic model	K_0_ (mol/(L.min)) R^2^	0.02 ± 0.003 0.8175	0. 22 ± 0.001 0.8676	0.25 ± 0.037 0.8506	0.25 ± 0.035 0.8593	0.25 ± 0.037 0.8490
First-order kinetic model	K_1_ (min^−1^) R^2^	0.05 ± 0.001 0.60335	0.05 ± 0.001 0.6589	0.05 ± 0.001 0.6639	0.04 ± 0.010 0.71437	0.04 ± 0.009 0.7160
Second-order kinetic model	k_2_ (L/mol min) R^2^	0.01 ± 0.001 0.9800	0.01 ± 0.001 0.9757	0.02 ± 0.002 0.9703	0.03 ± 0.002 0.9681	0.15 ± 0.028 0.7724

**Table 4:** Comparison of the efficiency of the current photocatalysts with the literature values for iron oxide and/or zeolite-based photocatalysts [46,47,48,49,50,51,52,53].

Catalyst	Light Source	Irradiation Time (min)	Dye Removal%	Ref.
ZnO/Fe_3_O_4_/g-C_3_N_4_	Visible	150	98	[46]
Au-zeolite	Sunlight	11	50	[47]
Ni-doped α-Fe_2_O_3_	Visible	140	86	[48]
Fe_2_O_3_/graphene oxide	UV	100	100	[49]
Fe_2_O_3_/Cu_2_O	UV	120	91	[50]
MgAC-Fe_3_O_4_/TiO_2_	UV	60	100	[51]
C_3_N_4_/NiFe_2_O_4_	Visible	80	98	[52]
TiO_2_-zeolite	UV	60	93	[53]
Fe_2_O_3_-zeolite	Sunlight	5	96% @ 20 mg	Present work
		2/3	99% @ 60 mg	
		1/2	~100% @ 75 mg	

## Data Availability

The data presented in this study are available on request from the corresponding author.

## Supplementary Materials

The following are available online at https://www.mdpi.com/article/10.3390/nano12020235/s1, Figure S1: Showing the scheme experimental part for the photocatalytic measurements, Figure S2: EDX spectrum of (**a**) zeolite, (**b**) Fe_2_O_3_, and (**c**) Fe_2_O_3_-zeolite photocatalyst, Figure S3: COD removal% versus exposure time during MB photodegradation under sunlight, Table S1: Values of the crystallographic parameters of Fe_2_O_3_ and Fe_2_O_3_-zeolite photocatalysts.
