# Supplementary material for: Highly Efficient Photocatalyst Fabricated from the Chemical Recycling of Iron Waste and Natural Zeolite for Super Dye Degradation
Source: Nanomaterials (Basel). 2022 Jan 12;12(2):235. doi: 10.3390/nano12020235 (PMC8778937; doi:10.3390/nano12020235)
Supplement: Supplementary file 1 [file nanomaterials-12-00235-s001.zip › nanomaterials-1381687-supplementary.pdf]

nanomaterials

Supplementary data

Highly Efficient Photocatalyst Fabricated from the Chemical Recycling of Iron Waste and Natural Zeolite for Super Dye Degradation

Fatma Mohamed [1,2], Safwat Hassaballa [3], Mohamed Shaban [3,*] and Ashour M. Ahmed [1]

[1] Nanophotonics and Applications (NPA) Lab, Physics Department, Faculty of Science, Beni-Suef University, Beni-Suef 62514, Egypt; f_chem2010@yahoo.com (F.M.); ashour.elshemey@gmail.com (A.M.A.)
[2] Polymer Research Laboratory, Chemistry Department, Faculty of Science, Beni-Suef University, Beni-Suef 62514, Egypt
[3] Department of Physics, Faculty of Science, Islamic University in Madinah, Al-Madinah Al-Munawarah 42351, Saudi Arabia; safwat.hassaballa@iu.edu.sa
* Correspondence: mssfadel@aucegypt.edu

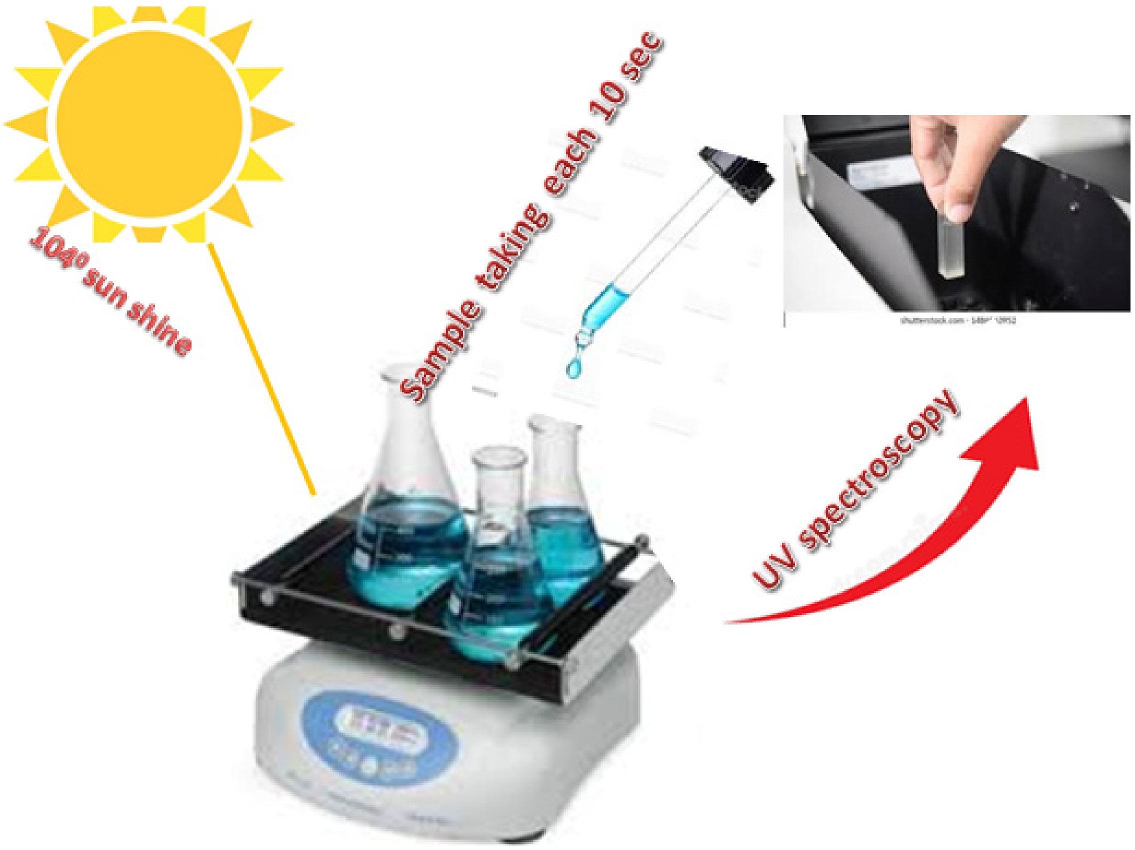

Figure S1. Scheme for the experimental photocatalytic measurements.

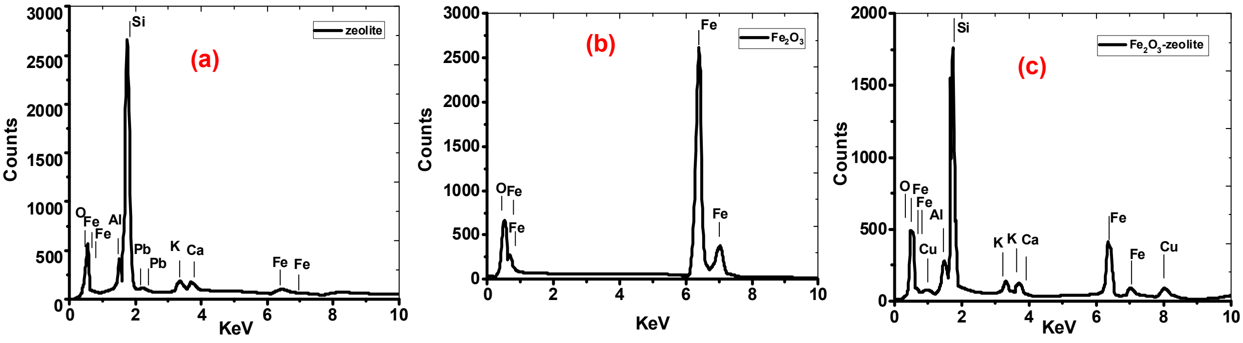

Figure S2. EDX spectrum of (**a**) zeolite, (**b**) Fe_2O_3, and (**c**) Fe_2O_3-zeolite photocatalyst.

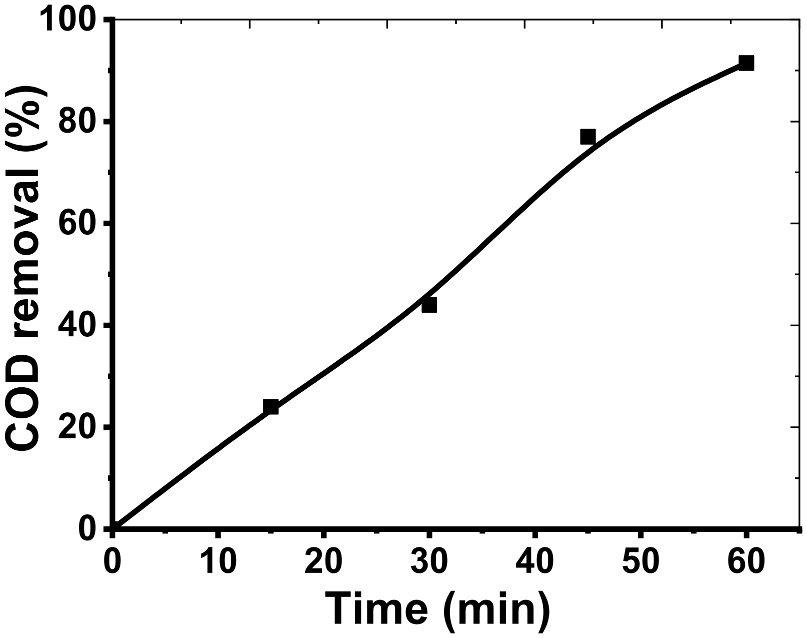

Figure S3. COD removal% versus exposure time during MB photodegradation under sunlight.

Table S1. Values of the crystallographic parameters of Fe_2O_3 and Fe_2O_3-zeolite photocatalysts.

Parameter	Fe_2O_3		Fe_2O_3-zeolite	
Planes [hkl]	[104]	[110]	[104]	[110]
Position (2θ °)	33.00	35.39	33.32	35.77
Height (cts)	92.86	83.37	40.83	27.45
d-spacing (nm)	0.2714	0.2536	0.2689	0.2510
Relative intensity (%)	100	89.78	100	67.22
Crystallite size (nm)	64.84	50.46	56.53	47.85
Microstrain (ε)	0.209	0.251	0.238	0.262
Dislocation δ $x10^{-4}$ (nm^{-2})	2.378	3.927	3.129	4.367
